# Supplementary material for: Impact of the COVID-19 Pandemic on the Incidence of Suicidal Behaviors: A Retrospective Analysis of Integrated Electronic Health Records in a Population of 7.5 Million
Source: Int J Environ Res Public Health. 2022 Nov 2;19(21):14364. doi: 10.3390/ijerph192114364 (PMC9654221; doi:10.3390/ijerph192114364)
Supplement: Supplementary file 1 [file ijerph-19-14364-s001.zip › ijerph-1963777-Supplementary File S1.pdf]

Impact of COVID-19 pandemic on the incidence of suicidal behaviors: a retrospective analysis of integrated electronic health records in a 7.5-million population

Supplementary File S1

CONTENTS

Supplementary methods 2

Table S1. Mental health resource utilization in Catalonia before and during the pandemic. 2

Table S2. Definition of the variables recorded at the Suicide Risk Code dataset used in the analysis.. 3

Table S3. Codes of the International Classification of Diseases (v10, clinical modification) considered for each mental health disorder 6

Supplementary figures 7

Figure S1. Decomposition of additive time series. 7

Figure S2. Monthly incidence, overall and according to gender; sub-analysis for individuals within the high (a), moderate (b), and low or very low (c) socioeconomic status. 8

Figure S3. Type of drug abuse among individuals with suicidal behaviors. Percentages for the pre-pandemic period (i.e., January 01, 2017 to February 29, 2020) and pandemic period (i.e., March 01, 2020 to June 30, 2022). 11

Supplementary TABLES.. 12

Table S4. Type of drug abuse of individuals with suicidal behaviors within the investigated period. Results are presented as no. and percentage of individuals.. 12

SUPPLEMENTARY METHODS

Table S1. Mental health resource utilization in Catalonia before and during the pandemic.

Year	Outpatient contacts	Annual change outpatients	Hospital contacts	Annual change hospitals
2018	1,601,576	-	25,828	-
2019	1,662,312	3.80%	23,346	-9.60%
2020	1,807,636	8.70%	23,573	1.00%
2021	1,884,922	4.30%	26,570	12.70%

Source: Annual healthcare reports – Catalan Health Service. Available at: https://catsalut.gencat.cat/ca/coneix-catsalut/presentacio/informes-memories-activitat/anual-catsalut/

Table S2. Definition of the variables recorded at the Suicide Risk Code dataset used in the analysis

Domain	Variable Code	Description
Patient	patient_id	Patient identifier
Patient	birth_date	Date of birth
Patient	gender	Gender
Patient	up_abs	Health area of the health centre associated with the patient
Patient	up_csm	Mental health centre associated with the patient
Patient	n_temptative	Number of suicide attempts registered at the Suicide Risk Code
Patient	exitus	Exitus
Patient	exitus_suicide_related	Exitus related with any suicide attempt registered at the SRC
Patient	exitus_date	Date of exitus
Patient	date	Date of inclusion of the patient in the SRC
Patient	extius_cause	Cause of exitus (in case of being related with the SRC)
Episode	patient_id	Patient identifier
Episode	episode_id	Episode identifier
Episode	num_temptativa	Number of the suicide attempt associated with the episode
Episode	date	Date of the episode
Episode	centre	Health centre associated with the episode
Episode	c1_death_thoughts	Answer to the first question of the MINI scale "Have you thought it would be better to die or have you wished you were dead?"
Episode	c1_points	Points associated with the answer to the first question. Yes: 1 point; No: 0 points.
Episode	c2_want_to_hurt	Answer to the second question of the MINI scale "Did you want to hurt yourself?"
Episode	c2_points	Points associated with the answer to the second question. Yes: 2 points; No: 0 points.
Episode	c3_suicide_thoughts	Answer to the third question of the MINI scale "Have you thought about suicide?"

Episode	c3_points	Points associated with the answer to the third question. Yes: 6 points; No: 0 points.
Episode	c4_suicide_planning	Answer to the fourth question of the MINI scale "Have you planned how to commit suicide?"
Episode	c4_points	Points associated with the answer to the fourth question. Yes: 10 points; No: 0 points.
Episode	c5_suicide_attempt	Answer to the fifth question of the MINI scale "Have you tried to commit suicide?"
Episode	c5_points	Points associated with the answer to the fifth question. Yes: 10 points; No: 0 points.
Episode	c6_previous_attemps	Answer to the sixth question of the MINI scale "Have you ever tried to commit suicide?"
Episode	c6_points	Points associated with the answer to the sixth question. Yes: 10 points; No: 0 points.
Episode	mini_points	Sum of the points obtained from the responses to the MINI scale screening
Episode	mini_risk_group	Risk group associated with the results from the MINI scale: Low (0 – 5 points), moderate (6 – 9 points), and high (≥ 10 points)
Episode	mental_disorder	The patient has a mental disorder present at the episode (Yes / No)
Episode	live_alone	The patient lives alone (Yes / No)
Episode	hopelessness	The patient has feelings of hopelessness (Yes / No)
Episode	agressiveness	The patient shows impulsiveness, aggressiveness, or an altered level of consciousness (Yes / No)
Episode	stressful_live_events	The patient has recently had any stressful life event such as unemployment, partner problems or family problems (Yes / No)
Episode	social_problems	The patient has recently had any social problems such as isolation, lack of support or socioeconomic problems (Yes / No)
Episode	lack_family	The patient has an absence of a family or social core (Yes / No)
Episode	painful_disease	The patient has a serious somatic or disabling illness with the presence of pain (Yes / No)
Episode	access_letal_means	The patient has access to lethal means (Yes / No)
Episode	alcohol	The patient is under the influence of the alcohol (Yes / No)

Episode	drugs	The patient is under the influence of any drug (Yes / No)
Episode	letality	Degree of lethality in case of attempt by clinical judgment (Mild / Moderate / Severe)
Episode	destination	Destination of the patient after the episode (Discharge at home / Hospital admission)
Episode	suicide_planning	There is suicidal ideation planning (Yes / No)
Episode	mental_disorder_code	Type DGN Catalog of Mental Disorder
Episode	mental_disorder_diagnostic	Diagnosis of mental disorder present at the episode
Episode	family_history_suicide	The patient has a family history of suicide (Yes / No)

Table S3. Codes of the International Classification of Diseases (v10, clinical modification) considered for each mental health disorder

F1*	Drugs
F10*	Alcohol abuse
F11*	Opioids abuse
F12*	Cannabis abuse
F13*	Hypnotic drugs abuse
F14*	Cocaine abuse
F17*	Tobacco abuse
F51*	Sleep disorders
F6*	Personality disorders
F31*	Bipolar disorders
F33* - F39*	Depression
F41*	Anxiety
F50*	Eating disorders
F20*, F25*	Schizophrenia
G30*	Alzheimer

SUPPLEMENTARY RESULTS

Figure S1. Decomposition of additive time series.

The observed rate was decomposed into a time series including the overall trend, the additive seasonal component, and random component using moving averages. The moving average considered a 12-month symmetric window with equal weights. The seasonal component was computed by averaging, for each time unit, over all periods. Finally, the error component is determined by removing trend and seasonal components from the original time series.

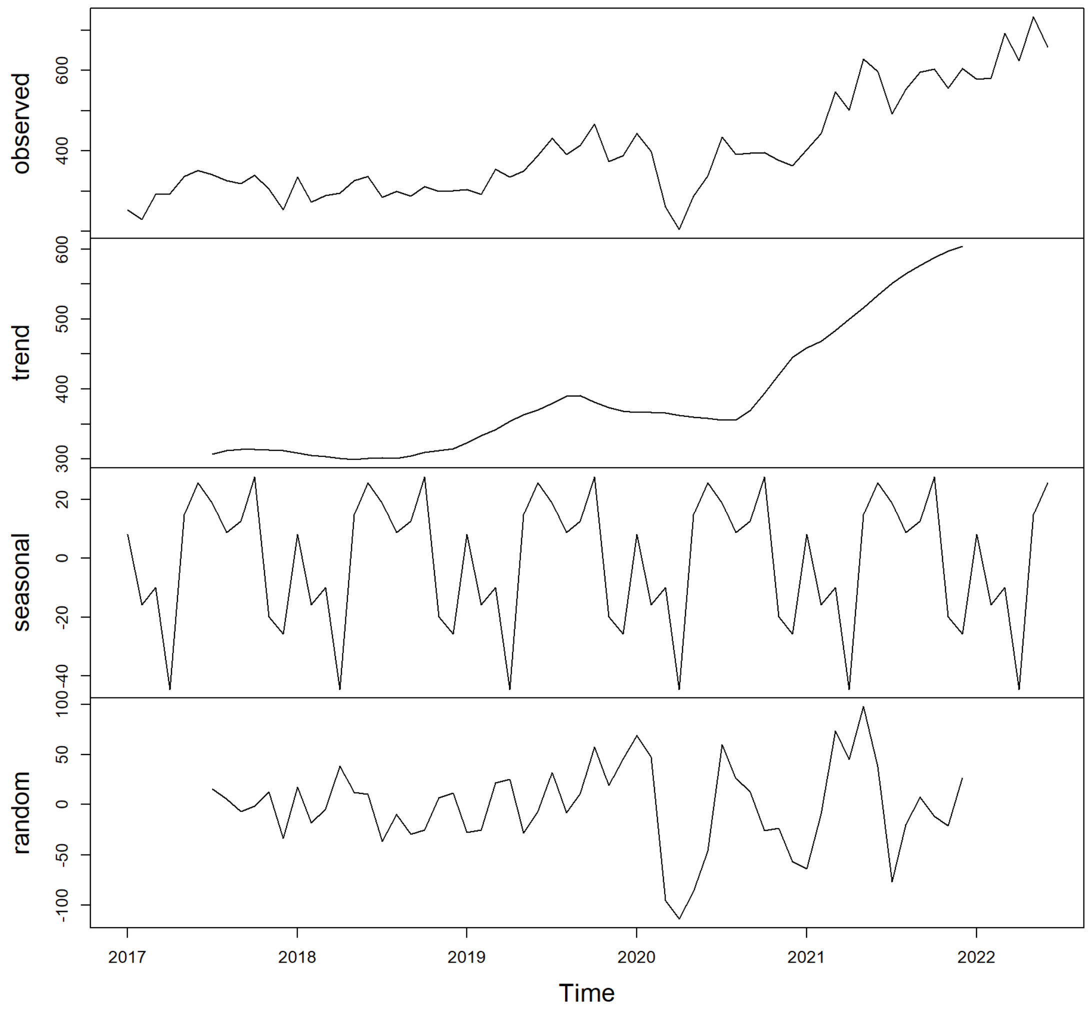

Figure S2. Monthly incidence, overall and according to gender; sub-analysis for individuals within the high (a), moderate (b), and low or very low (c) socioeconomic status.

a)

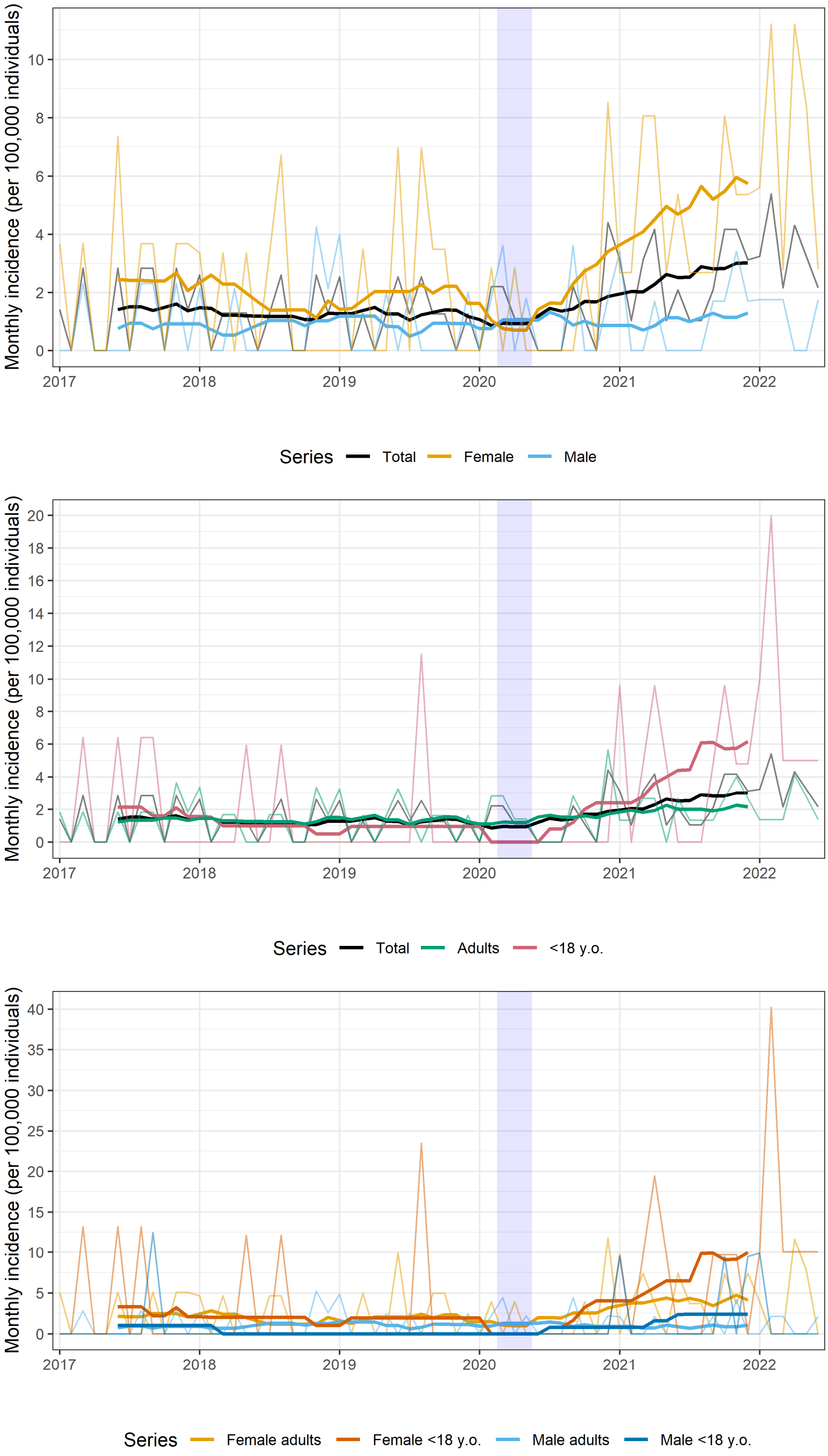

b)

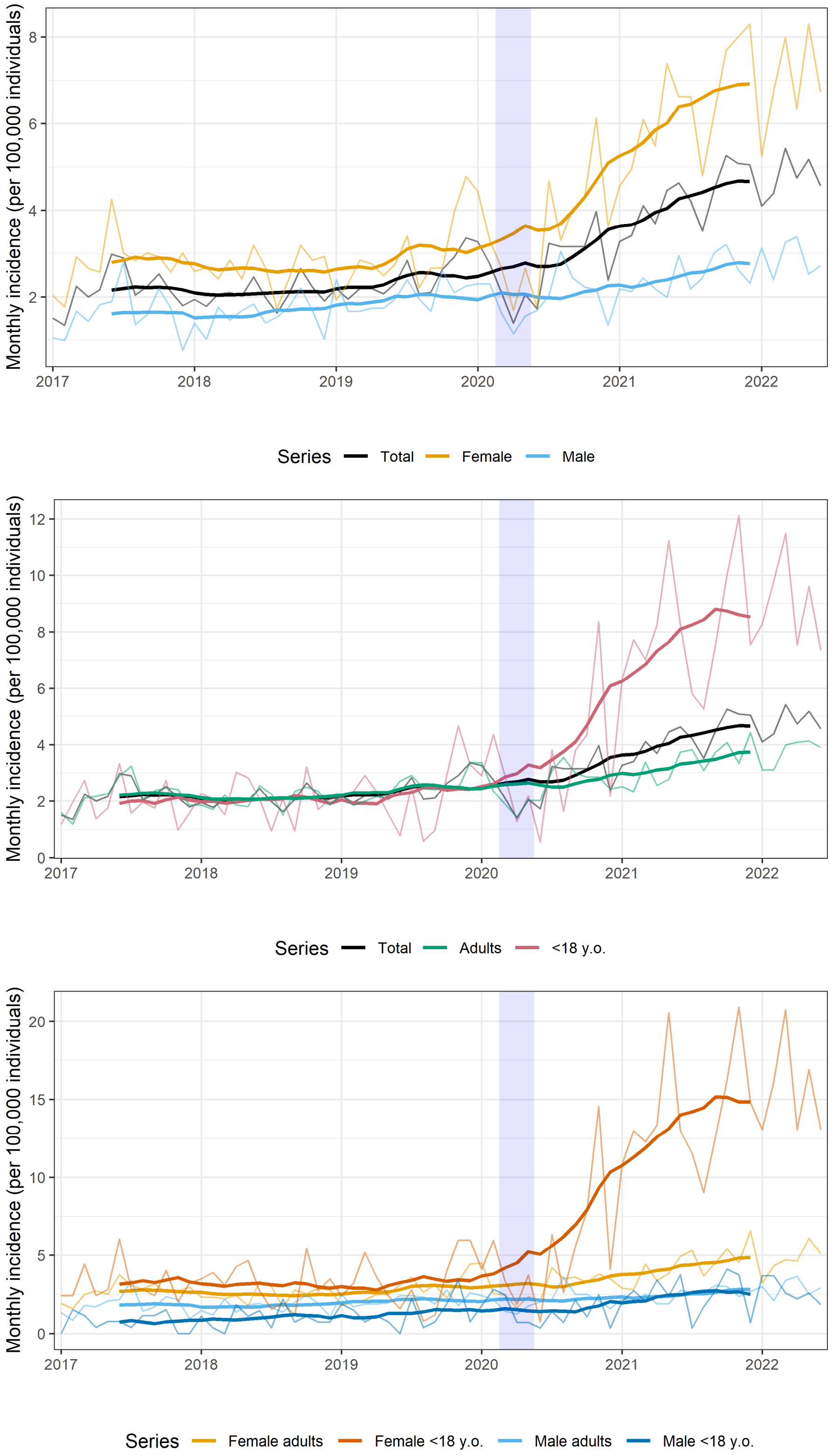

Monthly incidence (per 100,000 individuals)
2017
2018
2019
2020
2021
2022
Series
Total
Female
Male
Adults
<18 y.o.
Female adults
Female <18 y.o.
Male adults
Male <18 y.o.

c)

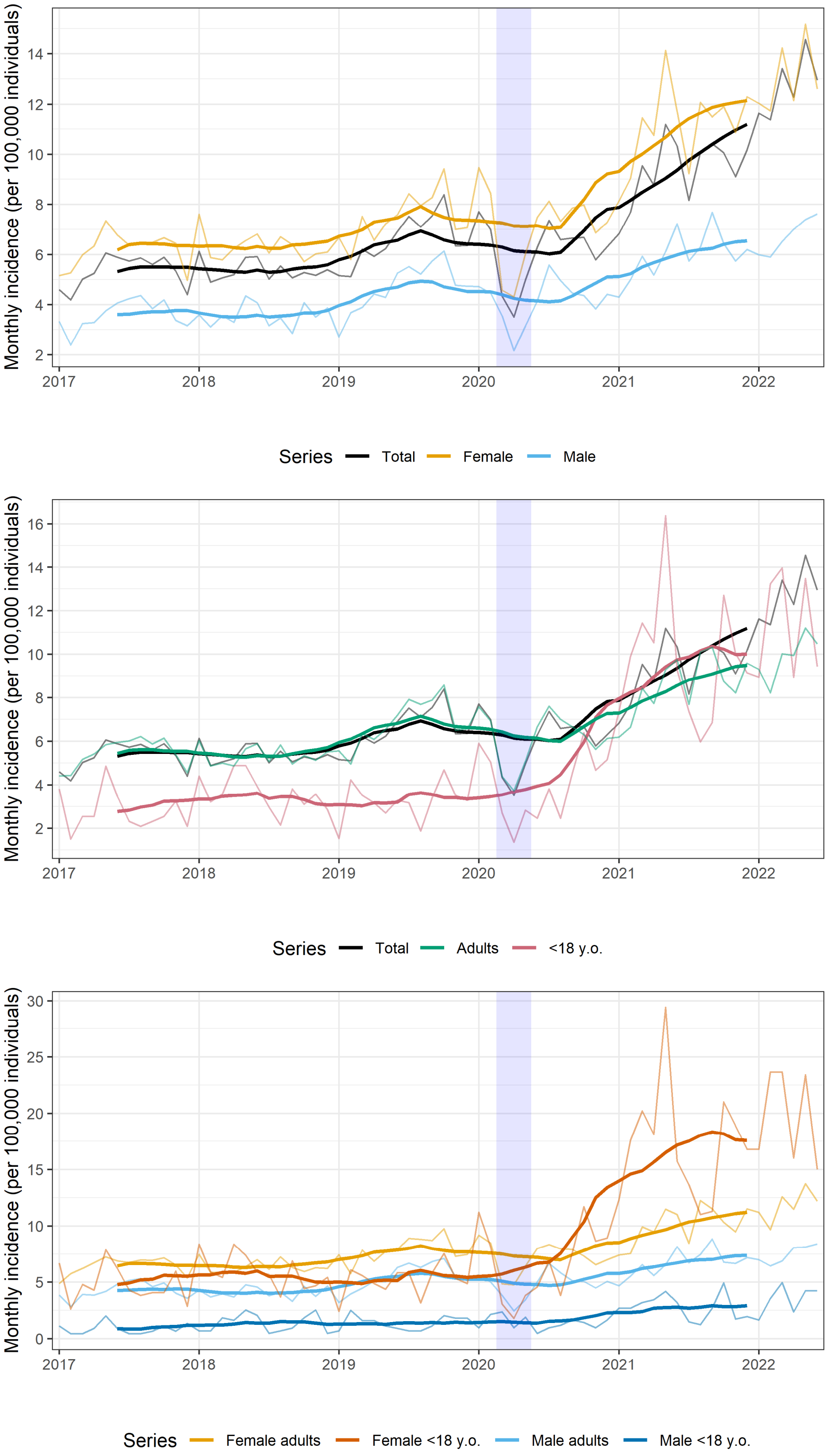
Monthly incidence (per 100,000 individuals)
2017
2018
2019
2020
2021
2022
Series
Total
Female
Male
Series
Total
Adults
<18 y.o.
Series
Female adults
Female <18 y.o.
Male adults
Male <18 y.o.

Figure S3. Type of drug abuse among individuals with suicidal behaviors. Percentages for the pre-pandemic period (i.e., January 01, 2017 to February 29, 2020) and pandemic period (i.e., March 01, 2020 to June 30, 2022).

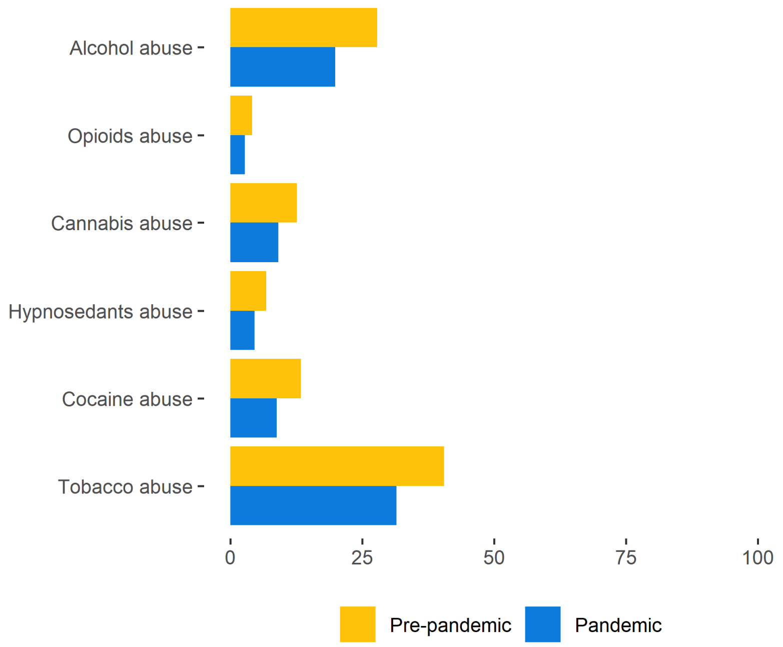

SUPPLEMENTARY TABLES

Table S4. Type of drug abuse of individuals with suicidal behaviors within the investigated period. Results are presented as no. and percentage of individuals.

	**2017** (N = 3641)	**2018** (N = 3638)	**2019** (N = 4489)	**2020** (N = 4291)	**2021** (N = 6528)	**2022**[1] (N = 3871)
Alcohol abuse	874 (24%)	773 (21.25%)	1033 (23.01%)	1002 (23.35%)	1149 (17.6%)	594 (15.34%)
Opioids abuse	132 (3.63%)	122 (3.35%)	160 (3.56%)	152 (3.54%)	163 (2.5%)	70 (1.81%)
Cannabis abuse	370 (10.16%)	346 (9.51%)	454 (10.11%)	456 (10.63%)	548 (8.39%)	265 (6.85%)
Hypnotic drugs abuse	180 (4.94%)	187 (5.14%)	258 (5.75%)	244 (5.69%)	275 (4.21%)	128 (3.31%)
Cocaine abuse	396 (10.88%)	393 (10.8%)	536 (11.94%)	430 (10.02%)	531 (8.13%)	272 (7.03%)
Tobacco abuse	1500 (41.2%)	1464 (40.24%)	1825 (40.65%)	1671 (38.94%)	1976 (30.27%)	1046 (27.02%)

[1] The observation period for year 2022 ends on June 30, 2022.
